# Identification and characteristics of a novel cecropin from the armyworm, *Mythimna separata*

**DOI:** 10.1186/s12866-020-01925-1

**Published:** 2020-08-01

**Authors:** Kaiqi Lian, Mingliang Zhang, Xiuli Liang, Lingling Zhou, Zhiqi Shi, Yajie Tang, Xueping Wang, Yuwei Song, Yuanchen Zhang

**Affiliations:** Henan Joint International Research Laboratory of Veterinary Biologics Research and Application, School of Biotechnology and Food Science, Anyang Institute of Technology, No.73 Huanghe Road, Anyang, Henan 225009 People’s Republic of China

**Keywords:** Antimicrobial peptide, Armyworm, Antimicrobial activity, Cecropin

## Abstract

**Background:**

The recent emergence of antibiotic-resistant strains of bacteria has increased the need to develop effective alternatives to antibiotics. Antimicrobial peptides have been considered as a promising product with several advantages.

**Results:**

In this present study, we identified a novel cecropin from the armyworm, *Mythimna separata* (armyworm cecropin 1, AC-1) by transcriptome sequencing and multi-sequence alignment analysis. The AC-1 precursor comprised 63 amino acid residues, containing a conserved cleavage site of the signal peptide, Ala_23_-Pro_24_, while the mature AC-1 included 39 amino acid residues. Chemically synthesized AC-1 exhibited low hemolytic activity against chicken red blood cells, low cytotoxicity against swine testis cells, and effective antimicrobial activity against *Salmonella*, *Escherichia coli*, *Klebsiella pneumonia*, and *Pseudomonas aeruginosa*. Its antimicrobial activity against *Salmonella* remained after incubation for 1 h at 100 °C or in 250 mM NaCl, KCl, or MgCl_2_ solution, implying good thermal- and salt-resistant stabilities. The bactericidal effect of AC-1 on *E. coli* gradually increased with increasing AC-1 concentration, resulting in deformation, severe edema, cytolysis, cell membrane damage, and reducing intracellular electron density. Additionally, recombinant AC-1 protein expressed in *E. coli* was digested by enterokinase protease to obtain AC-1, which showed similar antimicrobial activity against *E. coli* to chemically synthesized AC-1.

**Conclusions:**

This study identified a novel antimicrobial peptide that may represent a potential alternative to antibiotics.

## Background

The long-term overuse of conventional antibiotics has led to an increase in multidrug-resistant bacteria in animals and humans, including methicillin-resistant *Staphylococcus aureus* (MRSA), highlighting the need for useful alternatives to antibiotics for controlling and treating the resulting bacterial diseases [1]. Antimicrobial peptides (AMPs) are important components of natural immunity that are widely distributed in insects, mammals, amphibians, fish, plants, and bacteria [2–4]. AMPs exert activities against microorganisms, including bacteria, viruses, parasites, and fungi [5, 6], and have thus received much attention as potential antibiotic substitutes.

Researchers have accordingly discovered many novel AMPs in different species using various techniques, with the aim of identifying useful AMPs to substitute for traditional antibiotics to prevent and control bacterial infections. Insects do not display adaptive immunity but do possess an effective self-defense system similar to mammalian innate immunity [7, 8]. AMPs are important part of the insect defense system, and can directly kill pathogenic microorganisms or activate other immune pathways to clear pathogens [9]. Over 200 kinds of AMPs have been identified in insects to date, including about 30 kinds of cecropins. In the current study, we investigated a novel insect cecropin in the armyworm, *Mythimna separata*, named armyworm cecropin 1 (AC-1), and predicted and analyzed its physicochemical characteristics and structure. We also evaluated the hemolytic activities, cytotoxicity, and in vitro antimicrobial activities of chemically synthesized AC-1, and expressed recombinant AC-1 using and *Escherichia coli* prokaryotic expression system.

## Results

### Identification of AC-1

**Fig. 1:**
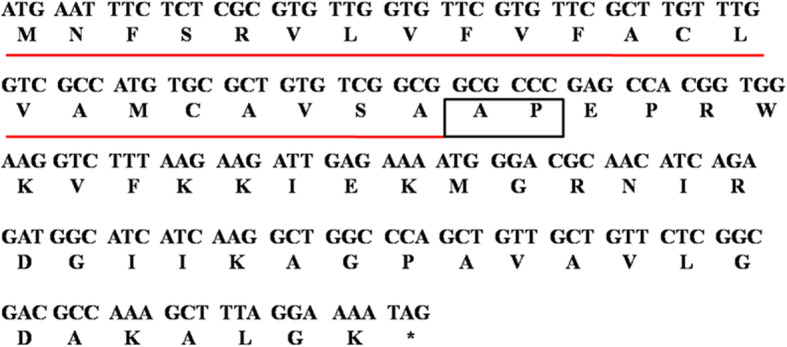
Nucleotide and amino acid sequences of AC-1 precursor. Underlined amino acid residues indicate the signal peptide; boxed amino acid residues indicate the cleavage site of the signal peptide; asterisk indicates the stop codon.

**Fig. 2:**
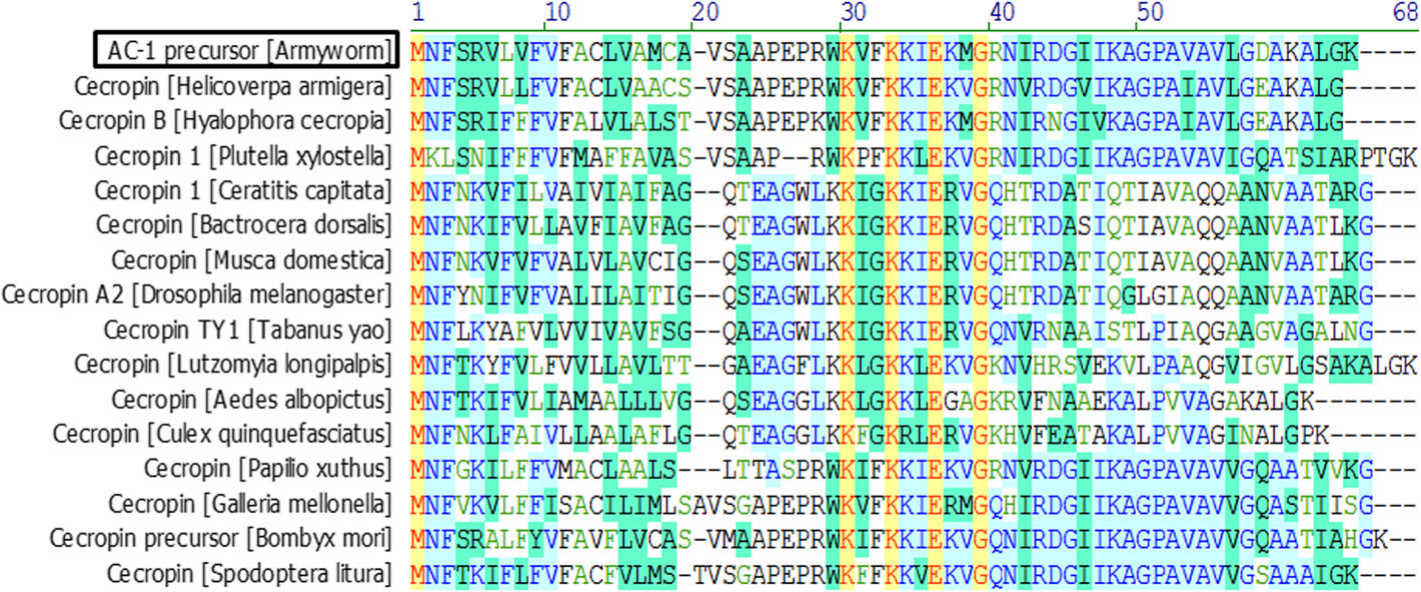
Multi-sequence alignment of AC-1 with representative cecropins from other insects. The conserved Ala-Pro cleavage site is boxed; identical residues are marked in yellow and highly conserved residues in blue.

Transcriptome sequencing of the armyworm was performed to obtain a gene pool, which was compared with the published AMP sequence. A novel cecropin was identified in the armyworm, and its gene and amino acid sequences are shown in Fig. 1. The gene sequence of the novel AMP was confirmed by polymerase chain reaction (PCR) amplification and gene sequencing. The open reading frame of the novel AMP was 192 bp in length and was translated into 63 amino acid residues. A conserved cleavage site was predicted in the signal peptide at Ala_23_-Pro_24 _using the SignalP-5.0 Server (http://www.cbs.dtu.dk/services/SignalP/). Multi-sequence alignment indicated that the cleavage site at Ala_23_-Pro_24 _was conserved among cecropins from many insects (Fig. 2). The mature peptide therefore comprised 39 amino acid residues, and was named armyworm cecropin-1 (AC-1). The amino acid sequence of AC-1 was highly similar to cecropins from other insects (Fig. 2).

### Physicochemical characteristics and secondary structure of AC-1

**Fig. 3:**
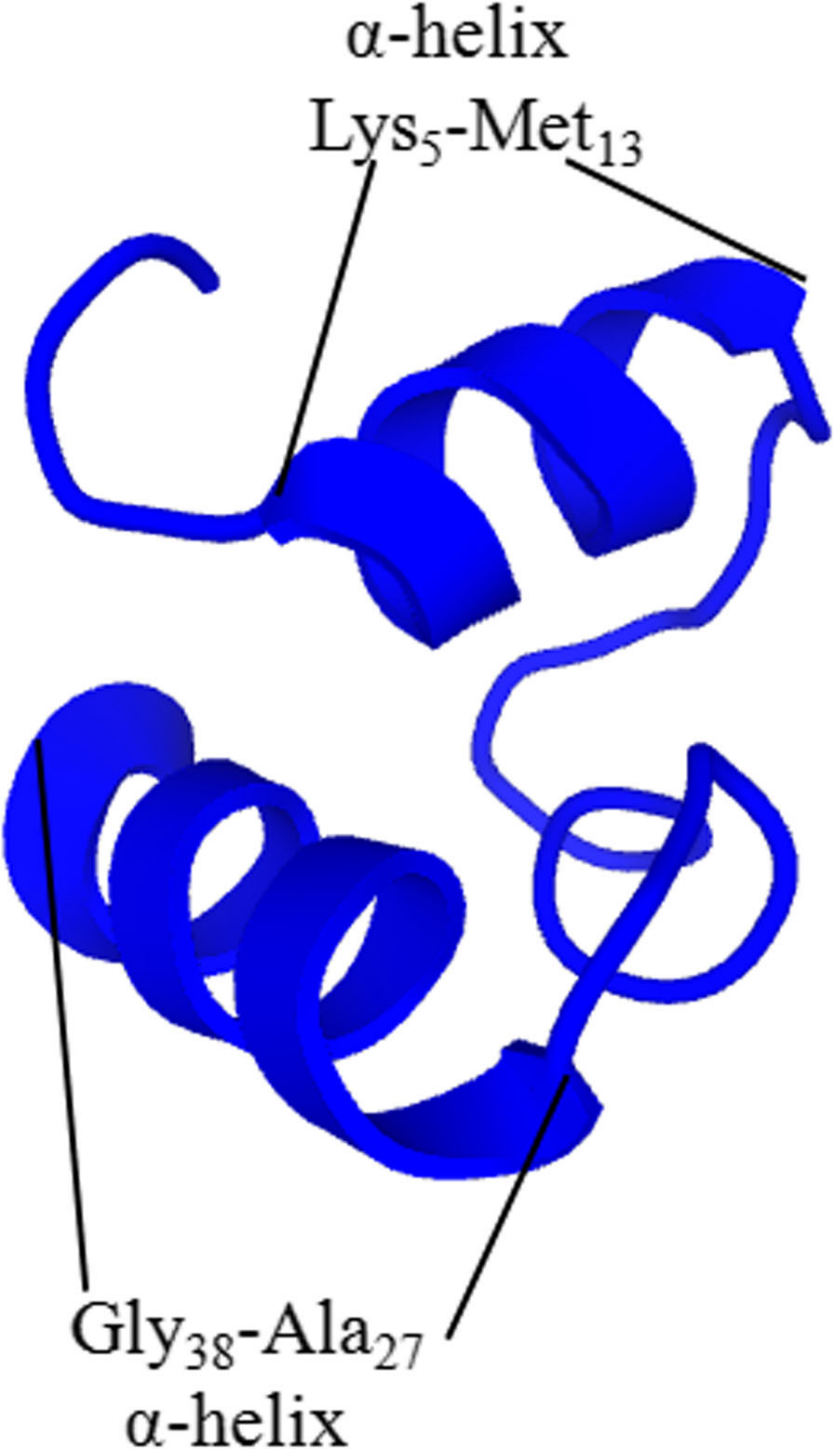
Secondary structure of AC-1 predicted by the online software PEP-FOLD3.5.

The physicochemical characteristics of AC-1 were predicted and shown in Table 1. AC-1 included 22 kinds of basic amino acid residues, and its secondary structure predicted using the online PEP-FOLD3 software suggested that AC-1 might adopt two α-helix conformations (Lys_5_-Met_13_, and Ala_27_-Gly_38_) (Fig. 3). The predicted secondary structure indicated an α-helix content of AC-1 of 58.97%, consistent with the predicted secondary structure of AC-1. We further analyzed the secondary structure of AC-1 by detecting and analyzing its circular dichroism (CD) spectrum in phosphate buffer saline (PBS) using a CD spectrometer (Chirascan; Applied Photophysics Limited, United Kingdom). AC-1 existed in three main structural forms in a physiological environment (20 mM PBS, pH 7.4): anti-parallel (44.5%), β-turn (22.6%), and random coil (32.6%) (Table 2).

**Table 1:** Physicochemical characterizations of the AC-1

Peptide	Grand average of hydropathicity	Number of amino acids (*n*)	Net charge	Theoretical pI	Molecular weight (Da)
AC-1	- 0.321	39	6+	10.38	4262.13

**Table 2:** Percentages of the secondary structural elements of AC-1 in PBS (20 mmol/L, pH 7.4)

	190–260 nm	195–260 nm	200–260 nm	205–260 nm	210–260 nm
Helix	6.9%	6.9%	7.6%	8.6%	10.7%
Anti-parallel	44.5%	40.3%	53.7%	52.1%	44.4%
Parallel	2.7%	3.6%	4.4%	4.8%	5.2%
Beta-Turn	22.6%	23.0%	24.7%	24.5%	22.1%
Random.Coil	32.6%	34.1%	31.6%	29.6%	33.5%
Total Sum	109.3%	107.9%	121.9%	119.6%	115.9%

### Hemolytic and cytotoxic activities of AC-1

**Fig. 4:**
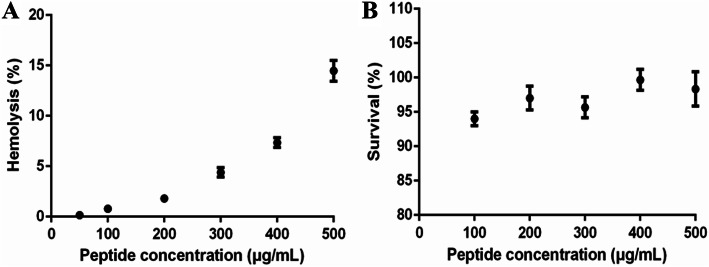
Hemolytic and cytotoxic activities of AC-1. **a**: Hemolytic activity of AC-1 evaluated in chicken red blood cells. **b**: Cytotoxic activity of AC-1 evaluated in swine testis cells.

The hemolytic activities of AMPs need to be assessed prior to their use in clinical practice. Chemically synthesized AC-1 showed low hemolytic capacity against chicken red blood cells, and the hemolysis rate was only 14.47 ± 1.03% even after treatment with AC-1 at 500 μg/mL for 1 h (Fig. 4a). The cytotoxicity of AC-1 was determined in swine testis (ST) cells. The viability of the ST cells was not significantly influenced by AC-1, and the cell survival rate remained > 90% even after treatment with at 500 μg/mL AC-1 for 1 h (Fig. 4b). These results indicated that AC-1 had low hemolytic and cytotoxic activities.

### Antimicrobial activity of AC-1

The antimicrobial activities of chemically synthesized AC-1 were evaluated *in vitro* by detecting the minimum inhibitory concentrations (MICs) using a standard two-fold broth microdilution method. AC-1 exhibited antimicrobial activities against standard strains of *Salmonella*, *E. coli*, and *Klebsiella pneumonia* with MICs of < 20.00 μg/mL, but did not inhibit *Bacillus cereus or Staphylococcus aureus* at 250.00 μg/mL (Table 3). AC-1 still showed antimicrobial activities against clinically isolated *Salmonella* and *E. coli* with MICs of 31.25 and 25.00 μg/mL, respectively (Table 3).

**Table 3:** Minimum inhibitory concentration (μg/mL) of the AC-1 and ampicillin against microorganisms

Microorganisms		MIC (μg/mL)
		AC-1 Ampicillin
Gram negative bacteria	*Escherichia coli* ATCC25922	7.80 (1.83 μmol/L) 0.50
	*Escherichia coli* clinical strain	25.00 (5.87 μmol/L) > 250.00
	*Salmonella* ATCC13076	12.50 (2.93 μmol/L) 1.00
	*Salmonella* clinical strain	31.25 (7.33 μmol/L) > 125.00
	*Klebsiella pneumonia* ATCC27853	15.63 (3.67 μmol/L) -
	*Pseudomonasaeruginosa* ATCC700603	125.00 (29.33 μmol/L) > 62.50
Gram positive bacteria	*Bacillus cereus* ATCC11778	> 250.00 (58.66 μmol/L) -
	*Staphylococcus aureus* ATCC29213	> 250.00 (58.66 μmol/L) 0.50

>, no activity detected at the concentration indicated. -, not assayed. Gram positive bacteria

### Thermal- and salt-resistant stabilities of AC-1

**Fig. 5:**
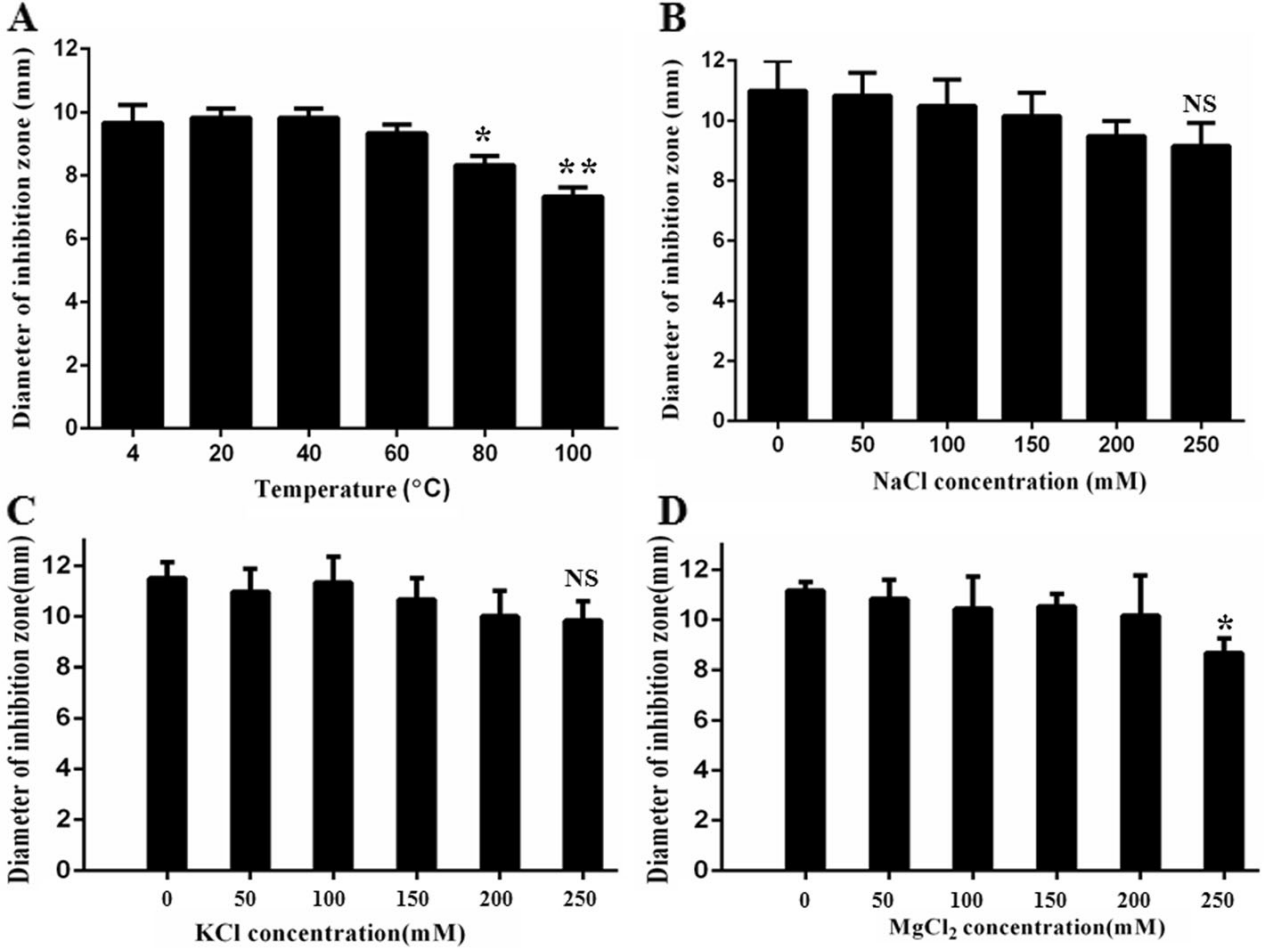
Thermal- and salt-resistant stabilities of AC-1. **a**: Thermal-resistant and (**b**-**d**) salt-resistant stabilities of AC-1 determined by detecting the antimicrobial activities of AC-1 against *Salmonella* (ATCC13076). *p < 0.05and**p < 0.01, compared with treatment at 40 °C or 0 mM MgCl_2_. NS indicates no significance, compared with the control.

We further evaluated the thermal- and salt-resistant stabilities of AC-1 by exposure to different temperatures, and NaCl, KCl, and MgCl_2_ concentrations for 1 h. Treatment at temperatures ranging from 4 to 60 °C had no obvious influence on the antimicrobial activities of AC-1 against *Salmonella*; however, its activity was lower at 80 °C and 100 °C, compared with 4 °C (Fig. 5a). The antimicrobial activities of AC-1 against *Salmonella* decreased with increasing NaCl, KCl, and MgCl_2_ concentrations from 0 mM to 250 mM, but the ability of AC-1 to inhibit *Salmonella* growth was not significantly decreased even at final concentrations of 250 mM NaCl, 250 mM KCl, and 200 mM MgCl_2_ for 1 h (Fig. 5b-d). These results suggested that AC-1 had good thermal- and salt-resistant stabilities.

### Antibacterial effect of AC-1 on *E. coli*

**Fig. 6:**
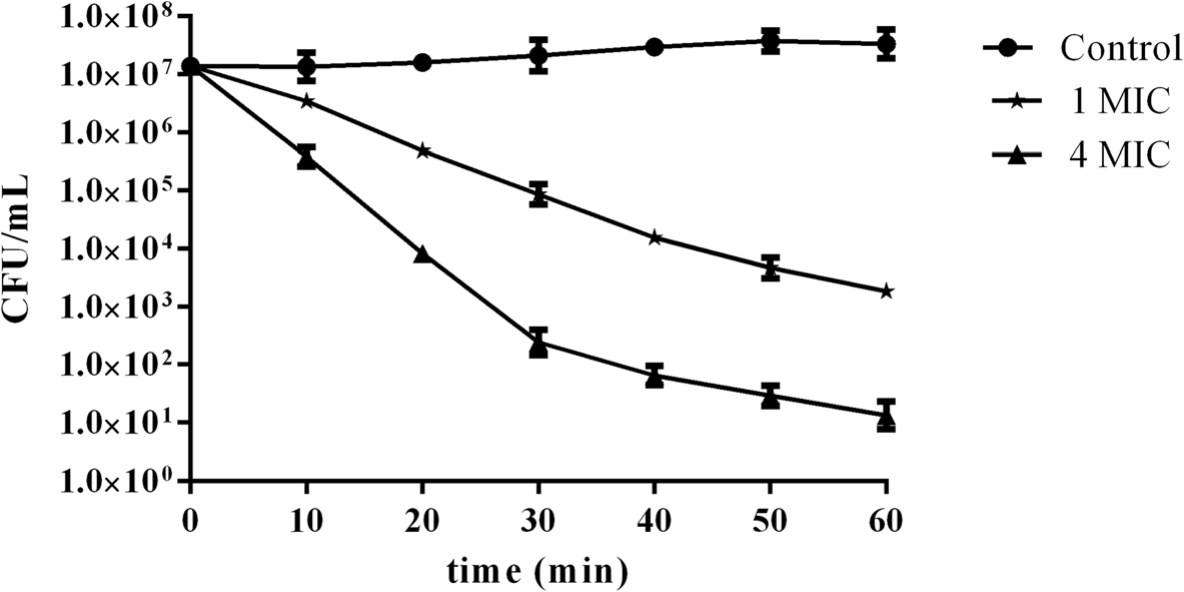
Time killing curve of AC-1 against *E. coli.*

**Fig. 7:**
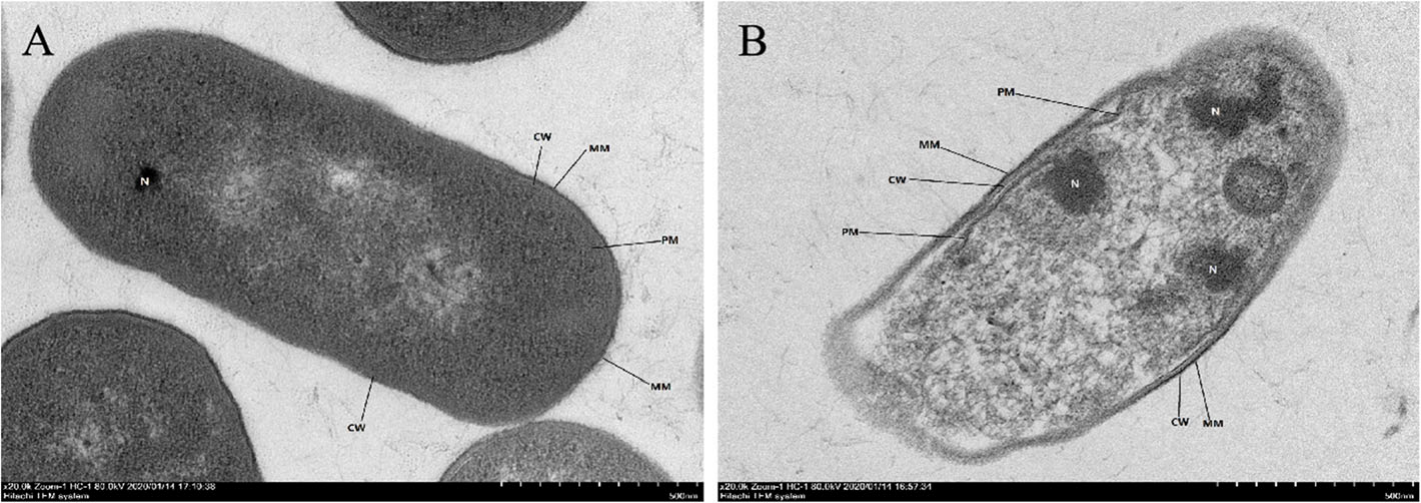
Transmission electron microscopy observation of *E. coli* treated with 0.9% NaCl solution (a) or 4 MIC AC-1 (b).

In order to further study antibacterial activities of AC-1, the time killing curve of AC-1 against *E. coli* was determined by the plate count method. The results showed that the bactericidal effect of AC-1 on *E. coli* gradually increased with increasing AC-1 concentration, and with increasing the action time within 60 min (Fig. 6). Transmission electron microscopy (TEM) observation suggested that AC-1 resulted in significant deformation, severe edema, cytolysis, cell membrane damage of *E. coli* compared with the control group, together with decreased intracellular electron density (Fig. 7). These results indicated that AC-1 showed effectively antibacterial activity against *E. coli.*

### AC-1 expression in *E. coli*

**Fig. 8:**
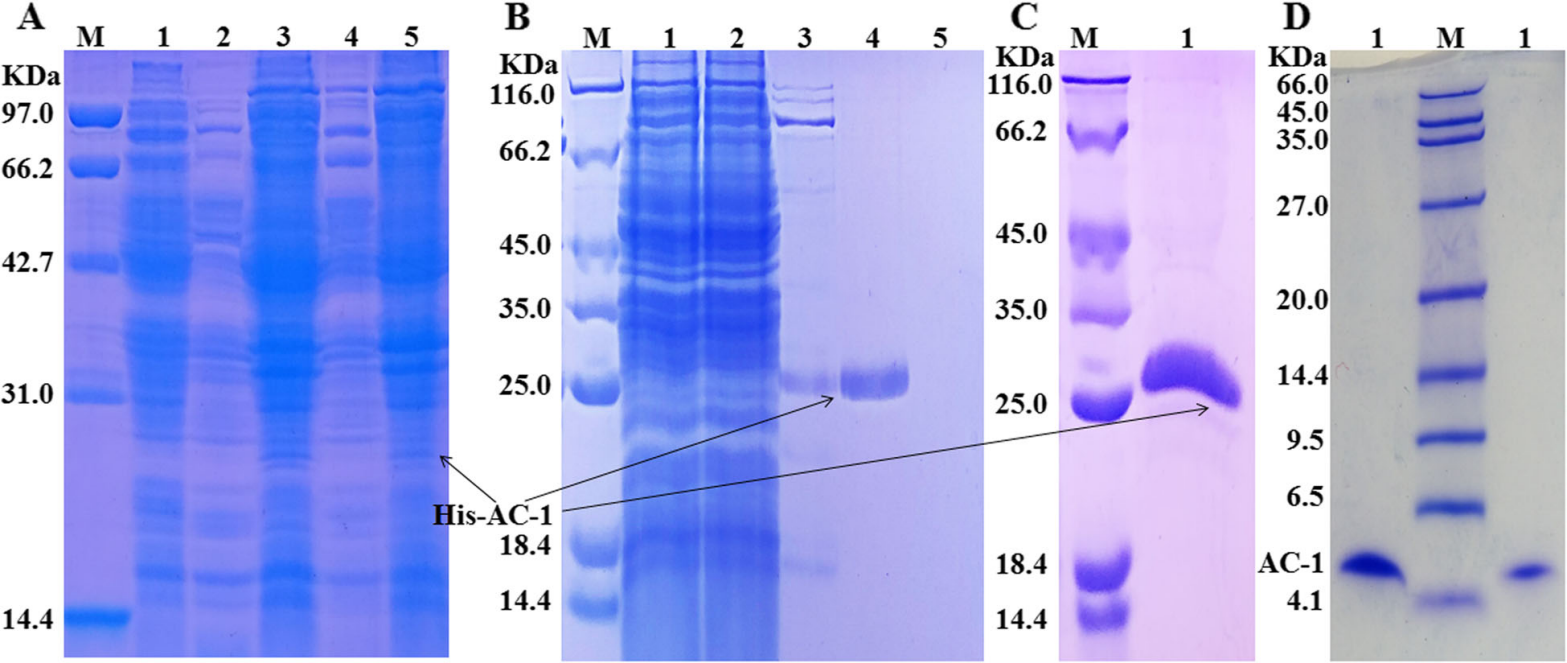
Expression and purification of recombinant AC-1 in *E. coli*. **a**: Expression and solubility of recombinant AC-1 in *E. coli*. *Lane* M, molecular weight marker; *lane* 1, *E. coli*bl21 (DE3)/pET-32a (+); supernatant (*lane* 2) and precipitate (*lane* 3) from *E. coli*bl21 (DE3)/pET-32a (+)-AC-1 induced at 20 °C; supernatant (*lane* 4) and precipitate (*lane* 5) from *E. coli*bl21 (DE3)/pET-32a (+)-AC-1 induced at 37 °C. **b**: Optimization of purification conditions for recombinant AC-1. *Lane* M, molecular weight marker; *lane* 1, treated inclusion body lysate; *lane* 2, flowthrough through an Ni-NTA gravity column; *lane* 3, solution eluted using 20 mmol/L imidazole eluent; *lane* 4, solution eluted using 50 mM imidazole eluent; *lane* 5, solution eluted using 200 mmol/L imidazole eluent. **c**: Purification of recombinant AC-1. *Lane* M, molecular weight marker; *lane* 1, purified recombinant protein AC-1 through Ni-NTA gravity column. **d**: Tricine-SDS-PAGE analysis of AC-1. *Lane* M, molecular weight marker; *lane* 1 and 2, purified AC-1 after recombinant protein AC-1 was digested by enterokinase

The chemical synthesis of peptide is expensive, and we therefore examined the expression of AC-1 in *E. coli* using recombinant DNA technology. The results showed that recombinant AC-1 was mainly expressed in inclusion bodies at 20 and 37 °C (Fig. 8a). Recombinant AC-1 was purified using a Ni-NTA gravity column with imidazole eluent (50 mM imidazole) (Fig. 8b-c). However, determination of the MIC of recombinant AC-1 suggested that it had no antibacterial activity against *E. coli*. Therefore, recombinant AC-1 was digested by enterokinase to produce AC-1 (Fig. 8d), which showed a MIC of 7.8 μg/mL against *E. coli* ATCC 25922. These results indicated that AC-1 could be prepared by an *E. coli* prokaryotic expression system.

## Discussion

Several methods can be used to identify novel AMPs. Qi et al. found two novel AMPs in the frog *Odorrana livida* by PCR amplification, using primer pairs based on the highly conserved sequence of known cathelicidins [10]. Zhou et al. identified five novel AMPs from *Hylarana guentheri* by isolation and purification [11], and Ma et al. identified 34 AMPs from *Rana nigrovittata* by peptidomics and genomics [12]. Yang et al. also identified a novel cathelicidin from the Chinese giant salamander *Andrias davidianus* using transcriptome sequencing and PCR amplification [13]. In the current study, we identified a novel cecropin from the armyworm by transcriptome sequencing and sequence alignment analysis. Sequencing of the PCR-amplified product confirmed the identity of the novel armyworm cecropin gene. Developments in high-throughput sequencing techniques have led to the discovery of increasing amounts of genetic data among which underlying AMPs are being increasingly identified.

Multi-sequence alignment and biological software analysis showed that the amino acid sequence of AC-1 was highly homologous to cecropins from other insects, and its precursor included the conserved signal peptide Ala-Pro cleavage site upstream of the mature peptide [14, 15]. In addition, AC-1 included the RWK and FKKIE(L) KVG structural domains that are conserved in cecropins from lepidopterous insects. Cecropins usually have a small molecular mass, positive charge, and α-helix conformation [15]. The physicochemical characteristics and predicted secondary structure of AC-1 were consistent with those of cecropins. The theoretical pI value of AC-1 was 10.38, which was > 9, suggesting that AC-1 would have a positive charge under physiological conditions [10].

In this study, we evaluated the antimicrobial, hemolytic, and cytotoxic activities, and the thermal- and salt-resistant stabilities of chemically synthesized AC-1. However, chemically synthesized AC-1 peptide is currently expensive, and may have reduced antimicrobial activities. It is therefore necessary to develop an efficient method for producing clinically useful peptides using a prokaryotic or eukaryotic expression method. Wang et al. produced the cecropin pxCECA1 in *E. coli* [15], while Pei et al. generated the antimicrobial peptide MDAP-2 using an *E. coli* prokaryotic expression system [16]. Li et al. expressed the peptide CGA-N46 in *Bacillus subtilis* DB1342 [17]. Luiz et al. produced the abaecin peptide [18] and Li et al. expressed the antimicrobial peptide fowlicidin-2, both in *Pichia pastoris* [19]. In the current study, we expressed recombinant AC-1 using an *E. coli* prokaryotic expression system, and the AC-1 obtained by enterokinase digestion exhibited similar antimicrobial activity to chemically synthesized AC-1.

## Conclusions

In conclusion, we identified a novel cecropin AC-1 from armyworms, which showed effective antimicrobial activities and low hemolytic and cytotoxic activities, as well as good thermal- and salt-resistant stabilities, implying that AC-1 may be clinically useful for preventing and treating bacterial diseases. We also successfully prepared AC-1 using an *E. coli* prokaryotic expression system and enterokinase digestion. Further studies are needed to determine the antimicrobial mechanisms and *in vivo* antimicrobial activities of AC-1.

## Methods

### Identification of antimicrobial peptide

Third instar larvae of the armyworm *Mythimna separata* were ground in liquid nitrogen and total RNA was extracted for transcriptomesequencing. The known AMP gene sequence was selected to align the transcriptomesequence using MegAlign software. The primers were designed based on the armyworm gene acquired by sequence alignment (sense primer: 5′-TTTGAATTAAGAACAAT-3′; antisense primer: 5′-CTATTTTCCTAAAGCTT-3′). The gene was amplified by PCR using the above primers with Premix LA Taq (Takara, Dalian, China) according to the manufacturer’s instructions. The PCR conditions were as follows: denaturation at 94 °C for 4 min, 36 cycles of denaturation at 94 °C for 40 s, annealing at 57 °C for 35 s, and elongation at 68 °C for 25 s, and a final elongation at 68 °C for 8 min. The PCR-amplified products were cloned into the pMD18-T vector (Takara) and positive plasmids were sequenced.

### Multi-sequence alignment of cecropins from different insects

The amino acid sequence of the AC-1 precursor was derived from the nucleotide sequence and subjected to multi-sequence alignment with the respective cecropins of different insects from the protein database at the National Center for Biotechnology Information (NCBI, https://www.ncbi.nlm.nih.gov/protein/?term=cecropin) using Vector NTI Advance® 11.5.3 software.

### Physicochemical characteristics and structure prediction of AC-1

The physicochemical characteristics of AC-1 were predicted by the ExPASy Bioinformatics Resource Portal (http://www.expasy.org/tools/) and its secondary structure was predicted using a novel online computational framework PEP-FOLD3.5 (http://bioserv.rpbs.univ-paris-diderot.fr/services/PEP-FOLD3/) [20]. The secondary structural components of AC-1 were calculated using an online SOPMA secondary structure prediction method (https://npsa-prabi.ibcp.fr/cgi-bin/npsa_automat.pl?page=npsa_sopma.html).

We further analyze the secondary structure of AC-1 by examining its CD spectrum, (0.2 mg/mL) in 20 mM PBS (pH 7.4) using a CD spectrometer (Chirascan, Applied Photophysics Limited, United Kingdom). The CD spectrum of AC-1 was recorded between 190 and 260 nm at 1 nm intervals at room temperature, with a 0.5 s response time and 1.0 nm step size.

### Hemolytic and cytotoxic activities of AC-1

AC-1 (purity > 98%) was synthesized by Shanghai Gil Biochemical Co., Ltd., China, purified by reverse high-performance liquid chromatography (Figure S1), and detected by mass spectrometry (Figure S2). Its hemolytic activity was tested using blood drawn from chickens and treated with sodium citrate anticoagulant. The treated blood was centrifuged at 3000×*g* for 10 min and washed three times in PBS. The red blood cells were counted and diluted to 2 × 10^7^/mL, and 100 μL of red blood cell suspension was mixed with 100 μL of different concentrations of AC-1 (final concentrations: 50, 100, 200, 300, 400, and 500 μg/mL). Triton X-100 solution was as a positive control and PBS as a negative control. After incubation for 1 h at 37 °C, the mixture was centrifuged at 3000×*g* for 10 min and the absorbance of the supernatants was then detected at 405 nm (OD_405_). The hemolysis ratio was calculated by the formula: hemolysis ratio = (*A*_405peptide_ - A_405PBS_)/(A_405Triton_ - A_405PBS_) × 100%. Each experiment was repeated three times.

The cytotoxicity of AC-1 was evaluated using a CCK-8 cell counting kit (Vazyme, Nanjing, China) in ST cells as described previously, with minor modifications [21]. A total of 100 μL of cells (about 5 × 10^4^/mL) per well was added into 96-well cell-culture plates and incubated for 24 h at 37 °C. Different concentrations of AC-1 (final concentrations: 100, 200, 300, 400, and 500 μg/mL) were added to the cells with further incubation for 12 h at 37 °C, followed by the addition of 10 μL of CCK-8 reagent into each well. The cell-culture plates were incubated for 1 h at 37 °C and the absorbance was determined at 450 nm using an automatic microplate reader. Each experiment was repeated three times.

### Antimicrobial assay of AC-1

The antimicrobial activity of AC-1 was analyzed by determining the MIC against different bacteria, as described previously, with minor modifications [22]. Ampicillin was used as a positive control. The synthesized AC-1 was dissolved in PBS and added into 96-well microtiter plates at two-fold dilutions. All the bacterial strains were cultured in Luria-Bertani (LB) broth at 37 °C to exponential phase. The bacterialsolutions were diluted to 2 × 10^6^ colony forming units (CFUs)/mL and added to 96-well microtiter plates at 50 μL per well, followed by 100 μL of AC-1/bacteria solution with mixing, and incubated for 16 h at 37 °C. Resazurin (10 μL 6 mM) was then added to each well and incubated for a further 3 h and the color change was observed in each well. Ampicillin and kanamycin were used as positive controls and PBS and LB broth as negative controls. The MIC was recorded as the concentration of the peptide in the last well that remained blue.

### Thermal- and salt-resistant stabilities of AC-1

We evaluated the thermal- and salt-resistant stabilities of AC-1 by determining the antimicrobial activities of AC-1 against *Salmonella* according to the inhibition zone method. To assess its thermal-resistant stability, 1 mg/mL AC-1 was incubated for 1 h at 4, 20, 40, 60, 80, and 100 °C, respectively. To determine its salt-resistant stability, 1 mg/mL AC-1 was incubated for 1 h with 0, 50, 100, 150, 200, and 250 mM of NaCl, KCl, and MgCl_2_, respectively. *Salmonella* was cultured to exponential phasein LB broth at 37 °C and diluted to 2 × 10^9^ CFUs/mL. Diluted bacterial solution (100 μL) was then mixed with 100 mL of sterilized LB solid medium and poured into a sterile culture dish. After cooling, the culture dish was punched using a diameter-same hole punch. The treated AC-1 solution was added into each well. Ampicillin was used as a positive control and PBS as a negative control. The culture dishes were incubated at 37 °C for 12 h and the diameters of the inhibition zones were measured using Vernier calipers. Each experiment was carried out in triplicate.

### Time killing curve of AC-1 against *E. coli*

Time killing curve of AC-1 against *E. coli* was determined as described previously [23]. *E. coli* in logarithmic growth phase were centrifuged to collect the precipitate, diluted with sterile LB liquid medium to 2 × 10^7^ CFU/mL, followed by the addition and mixing of 400 μL of bacterial solution and 400 μL of AC-1 solution to final concentrations of AC-1 of 1 MIC and 4 MIC, respectively. NaCl solution was used as negative control. The mixed solution was incubated at 37 °C for 0, 10, 20, 30, 40, 50, 60 min, respectively, and then centrifuged to collect the bacteria. The bacterial precipitate was washed and suspended in PBS, and 50 μL of bacterial solution was serially diluted 10 times. Each dilution of bacterial solution (100 μL) was spread on LB plates and cultured for 14 h, and the number of bacteria was then calculated. Each experiment was carried out in triplicate.

### TEM

*E. coli* in logarithmic growth phase were centrifuged to collect the precipitate. The precipitate was washed three times with sterile PBS and diluted with sterile PBS to 2 × 10^7^ CFU/mL, and 600 μL of bacterial solution and 600 μL of AC-1 solution were thoroughly mixed, to give a final concentration of AC-1 of 4 MIC. The mixed was incubated in a water bath at 37 °C for 1 h. *E. coli* treated with NaCl solution was used as a negative control. The two groups of *E. coli* were fixed, dehydrated, and stained, as described previously [24], and examined by TEM (HT7700; Hitachi, Japan).

### Expression of AC-1 in *E. coli*

The recombinant AC-1 gene included 39 amino acid residues of the mature peptide AC-1 and the enterokinase cleavage site at 5′-terminus of AC-1 gene. AC-1 gene was synthesized and cloned into pET-32a (+) using the restriction enzymes *Kpn* I and *Hind* III. The recombinant plasmid pET-32a (+)-AC-1 was transformed into *E. coli* BL21 (DE3) to express recombinant AC-1 by isopropyl-β-D-thiogalactoside induction. The resulting recombinant protein was purified using a Ni-NTA gravity column as described previous [25], and then digested using enterokinase. The digested solution was passed through a Ni-NTA gravity column, and the flowthrough was collected, dialysed, and concentrated to obtain AC-1 as described previous [25].

### Statistical analysis

Data were analyzed using GraphPad Prism 6 software. A value of *p* < 0.05 was considered significant and *p* < 0.01 was considered highly significant.

## Supplementary information

**Additional file 1: Figure S1.** Purification of chemically synthesized AC-1 by reverse-high performance liquid chromatography.

**Additional file 2: Figure S2.** Detection of chemically synthesized AC-1 by mass spectrometry.

## Data Availability

All data generated or analyzed during this study are included in this published article and its supplementary information files.

## Abbreviations

*E.coli*: *Escherichia coli*
MRSA: Methicillin-resistant *Staphylococcus aureus*
AMP: Antimicrobial peptide
PCR: Polymerase chain reaction
CD: Circular dichroism
ST cell: Swine testis cell
MIC: Minimum inhibitory concentration
mM: mmol/L
TEM: Transmission electron microscopy
NCBI: National Center for Biotechnology Information
PBS: Phosphate buffer saline
CFU: Colony forming units
LB: Luria-Bertani
