# Supplementary figures and images for: Identification and characteristics of a novel cecropin from the armyworm, Mythimna separata
Source: BMC Microbiol. 2020 Aug 1;20:233. doi: 10.1186/s12866-020-01925-1 (PMC7395354; doi:10.1186/s12866-020-01925-1)

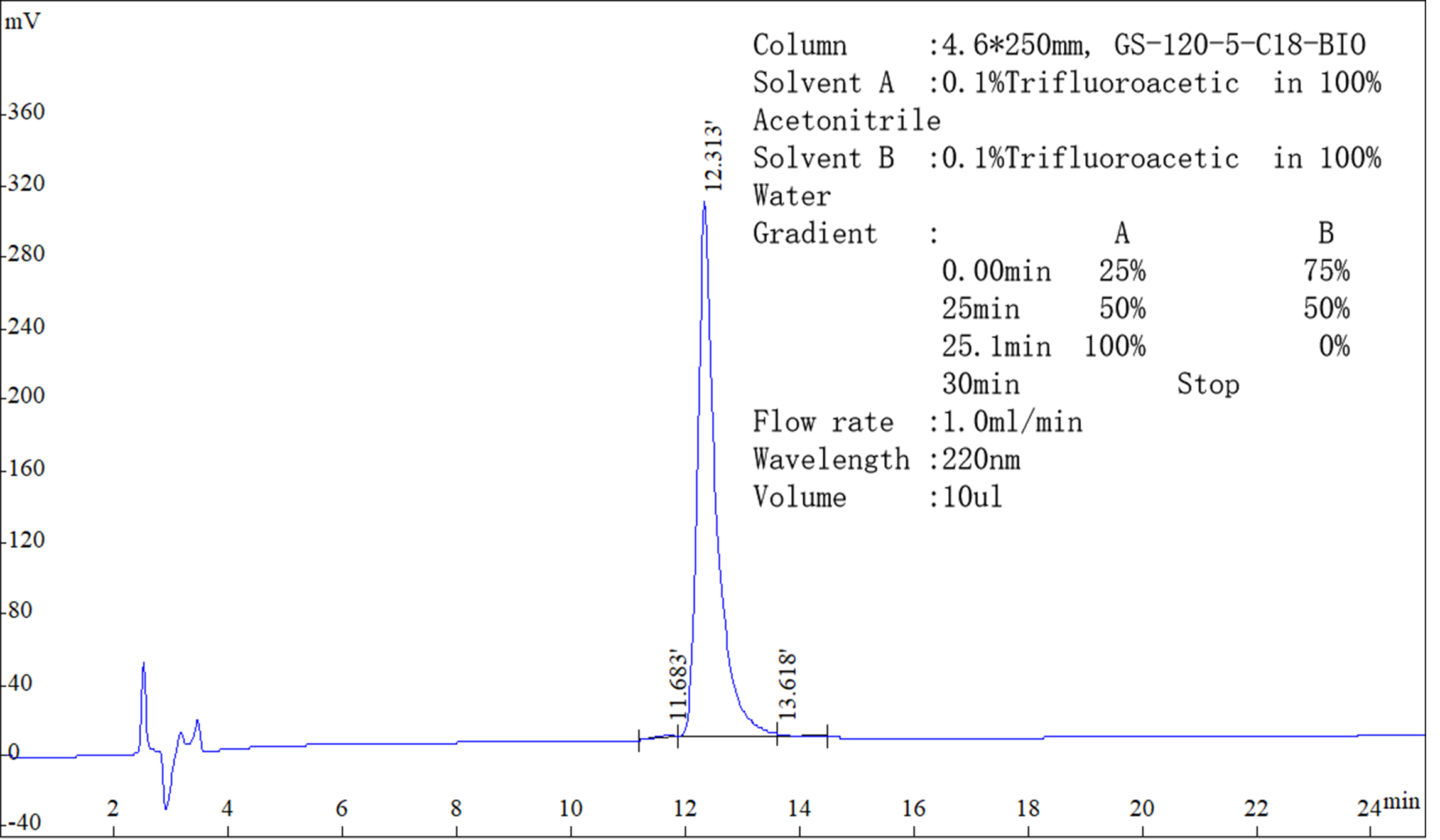

Supplement: Supplementary file 1 — Additional file 1: Figure S1. Purification of chemically synthesized AC-1 by reverse-high performance liquid chromatography. [file 12866_2020_1925_MOESM1_ESM.tif]

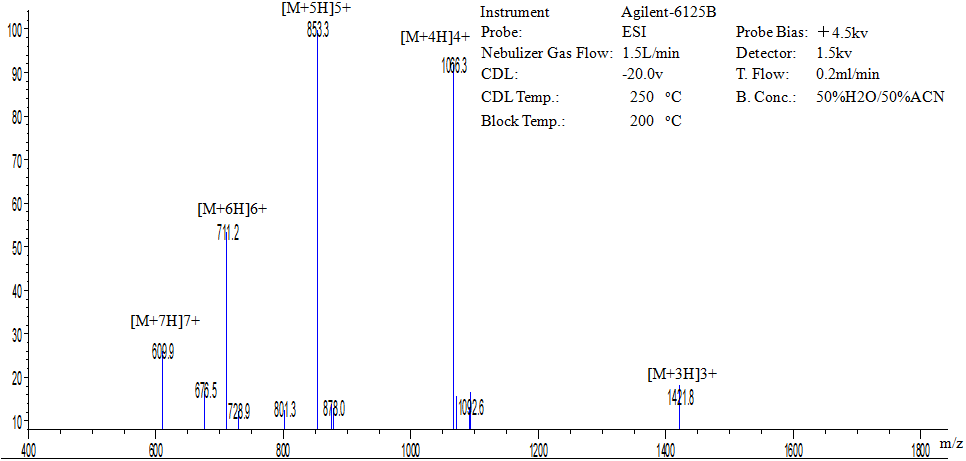

Supplement: Supplementary file 2 — Additional file 2: Figure S2. Detection of chemically synthesized AC-1 by mass spectrometry. [file 12866_2020_1925_MOESM2_ESM.tif]
